# Simulation and Investigation of 26% Efficient and Robust Inverted Planar Perovskite Solar Cells Based on GA_0.2_FA_0.78_SnI_3_-1%EDAI_2_ Films

**DOI:** 10.3390/nano12213885

**Published:** 2022-11-03

**Authors:** Hussein Sabbah, Jack Arayro, Rabih Mezher

**Affiliations:** College of Engineering and Technology, American University of the Middle East, Eqaila 54200, Kuwait

**Keywords:** solar cell, thin films, SCAPS simulation, tin-based perovskite, power conversion efficiency, electron transport layer

## Abstract

A hybrid tin-based perovskite solar cell with *p*-*i*-*n* inverted structure is modeled and simulated using SCAPS. The inverted structure is composed of PEDOT:PSS (as hole transport layer—HTL)/GA${}_{0.2}$FA${}_{0.78}$SnI${}_{3}$-1% EDAI${}_{2}$ (as perovskite absorber layer)/C60-fullerene (as electron transport layer—ETL). Previous experimental studies showed that unlike conventional tin-based perovskite solar cells (PSC), the present hybrid tin-based PSC passes all harsh standard tests and generates a power conversion efficiency of only 8.3%. Despite the high stability that this material exhibits, emphasis on enhancing its power conversion efficiency (PCE) is crucial. To that end, various ETL and HTL materials have been rigorously investigated. The impact of energy level alignment between HTL/absorber and absorber/ETL interfaces have been elucidated. Moreover, the thickness and the doping concentration of all the previously mentioned layers have been varied to inspect their effect on the photovoltaic performance of the PSC. The optimized structure with CuI (copper iodide) as HTL and ZnOS (zinc oxysulphide) as ETL scored a PCE of 26%, which is more than three times greater than the efficiency of the initial structure. The current numerical simulation on GA${}_{0.2}$FA${}_{0.78}$SnI${}_{3}$-1% EDAI${}_{2}$ could greatly increase its chance for commercial development.

## 1. Introduction

In the aim of reducing the use of traditional fossil fuels responsible for climate change and global warming, extensive work has been done on developing other sources that are environmentally safe, renewable, and sustainable. One of the rising sources of renewable energy is solar, which can be harnessed by solar cells [1,2]. Many generations of solar cells appeared, starting with silicon-based ones, which have a high manufacturing cost. Then, another generation of more accessible and affordable solar cells emerged: lead (Pb)-based perovskite solar cells (PSCs). Indeed, the latter type of cells possesses superior properties: high absorption coefficients [3,4], low exciton binding energies [5], long carrier diffusion lengths, high carrier mobility ,and tunable bandgaps [6], achieving a high-power conversion efficiency (PCE), reaching 24% [7]. Despite its advantages, the use of the latter type of cells is limited by the toxicity level of Pb. To overcome this drawback, studies [8,9,10] showed that Pb could be replaced by a less toxic element such as Sn, Bi, Ge, or Sb. Compared to the other alternative elements, Sn-based PSCs have shown very promising performance. However, the achieved performance is still lower than that of Pb-based analogues. The limited performance is mainly caused by the reduced formation energy of Sn vacancies (V${}_{Sn}$) and the low air-stability arising from the rapid oxidation of Sn${}^{2+}$ to Sn${}^{4+}$ [11].

Among Sn-based cells, the top performing one is found to be FASnI${}_{3}$, as it has lower conductivity and mobility than its MA and Cs counterparts, indicating that FASnI${}_{3}$ has decent stability in air and less tendency to produce V${}_{Sn}$ [12,13,14]. In addition, a study made by Koh et al. [15] on the p-type orthorhombic FASnI${}_{3}$ showed that these films have a bandgap of 1.41 eV. Previous works on the enhancement of FASnI${}_{3}$ [16,17] demonstrated promising PCE, reaching 25.94%, obtained by increasing the active layer thickness and tuning the concentration of both defect density and doping of the absorber. However, FASnI${}_{3}$ exhibits lower experimental PCE (9%) and significantly weak stability [18,19,20] compared to the Pb-based analogues.

To overcome the oxidation issue, extensive work has been done on improving the stability and the performance of tin-based PSC [21,22,23,24,25], by changing the electronic structure of perovskite, providing a uniform and close-packed film, introducing hydrogen bonding, or adopting a hydrophobic shell [22,23,24,26,27]. In addition, stable Sn-based perovskite devices can be obtained through an appropriate choice of organic cation to substitute a proportion of FA [27], ensuring an effective protection against water penetration. A study by Zhao et al. [28] suggested GA${}^{+}$ as an organic cation that can be used to produce FASnI${}_{3}$ or hybrid FA+/MA+ device with PCE of 4–8%. Moreover, applying large hydrophobic ammonium cations such as butylammonium (BA${}^{+}$) or phenylethylammonium (PEA${}^{+}$) within FASnI${}_{3}$ leading to the production of a quasi-2D [29] or hybrid 2D/3D [22,23,25] PSC was found to stabilize the device while providing a relatively improved PCE of 9.0% [22]. In addition, Jokar et al. incorporated guanidinium (GA${}^{+}$) as a nonpolar organic cation, in varied proportions into the formamidinium (FA${}^{+}$) tin triiodide perovskite (FASnI${}_{3}$) crystal structure in the presence of 1% ethylenediammonium diiodide (EDAI${}_{2}$) as an additive [30]. In this experimental study, it was found that the obtained GA${}_{0.2}$FA${}_{0.78}$SnI${}_{3}$-1% EDAI${}_{2}$ (guanidinium formamidinium tin iodide with 1% of ethylenediammonium diiodide; abbreviated as E1G20 in the remaining of the paper) with planar inverted structure scored a PCE of 9.6% while having extended stability.

Apart from the above-mentioned techniques and their positive impact on the stability and the performance of solar cells, the inverted structure *p*-*i*-*n* appears to provide improved properties compared to the conventional *n*-*i*-*p* structure. In fact, inverted structures *p*-*i*-*n* exhibit better stability and weak current density-voltage (J-V) hysteresis effect compared to regular designs [29,31,32,33,34]. Moreover, inverted structures surpass their counterparts in cost-effectiveness, roll-to-roll printing, low-temperature processing using a slot die coating, and compatibility with large area device fabrications [35].

This work aims to suggest possible optimization strategies to improve the efficiency of E1G20 solar cell, by studying various device parameters through the solar cell capacitance simulator (SCAPS-1D) [36], never been conducted so far, starting from the experimental results obtained by [30]. In this study, the engineering of HTL/perovskite and perovskite/ETL interfaces has been performed through the investigation of different HTL (hole transport layer) and ETL (electron transport layer) materials. Then, the effect of the doping concentration and the thickness of the absorber, ETL and HTL defect density and the thickness of the absorber layer and its doping concentration on the overall performance of the proposed device were considered. Finally, it has been proven that the inverted E1G20-based perovskite solar cells device can have a simulated power conversion efficiency PCE of 26%, which is the major contribution of this paper.

## 2. Materials and Methods

In this work, a numerical study was performed, considering E1G20 perovskite as the light absorber. The adopted simulation software was SCAPS 3.8, which is a 1D solar cell simulation software developed at the Department of Electronics and Information Systems (ELIS) of the University of Gent, Ghent, Belgium [36]. SCAPS allows simulation of multi-layer solar cells (up to seven layers). SCAPS allows calculating and observeingvarious electrical characteristics, such as PCE, hetero-junction energy band structure, current-density, open circuit voltage (V${}_{oc}$), short circuit current density J${}_{sc}$, quantum efficiency (QE), current density, and fill factor FF. SCAPS solves with an adapted algorithm, the Poisson’s equation, Equation (1) and the continuity equations of both charge carriers: the electron in Equation (2) and hole in Equation (3)

(1) $$\frac{d}{dx}\left(-\varepsilon \left(x\right)\frac{d\psi }{dx}\right)=q\left[p\left(x\right)-n\left(x\right)+N_{D}^{+}\left(x\right)+p_{t}\left(x\right)-n_{t}\left(x\right)\right]$$

(2) $$\frac{dp_{n}}{dt}=G_{p}-\frac{p_{n}-p_{n0}}{\tau_{p}}+p_{n}\mu_{p}\frac{d\xi }{dx}+\mu_{p}\xi \frac{dp_{n}}{dx}+D_{p}\frac{d^{2}p_{n}}{dx^{2}}$$

(3) $$\frac{dn_{p}}{dt}=G_{n}-\frac{n_{p}-n_{p0}}{\tau_{n}}+n_{p}\mu_{n}\frac{d\xi }{dx}+\mu_{n}\xi \frac{dn_{p}}{dx}+D_{n}\frac{d^{2}n_{p}}{dx^{2}}$$

**Figure 1:**
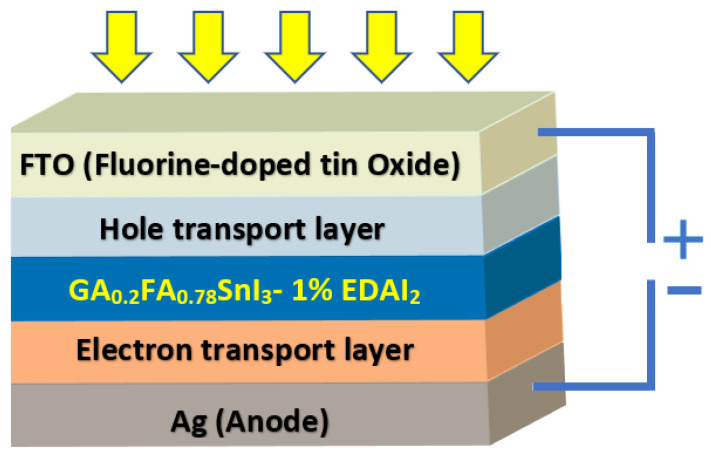
Schematic diagram of E1G20-based PSC structure.

The simulated device consists of a sequence of layers FTO/HTL/E1G20/ETL/Ag (FTO is fluorine-doped tin oxide), represented in Figure 1 and the simulation is carried out in the *p*-*i*-*n* configuration.

All simulations were performed at a temperature of 300K under standard illumination of 1000W/m${}^{2}$, and an air mass of AM1.5 G. The absorber layer (E1G20) is sandwiched between HTL and ETL layers. As a front contact FTO is used, while silver (Ag) is considered for the back metal contact material, as shown in Figure 1.

In the current study, the initial cell geometrical structure is extracted from an experimental work done by Jokar et al. featuring materials of common interest [30]. In Jokar’s work, the chosen absorber, HTL and ETL were E1G20, PEDOT:PSS (Poly(3,4-ethylenedioxythiophene)-poly(styrenesulfonate)) and C60 (fullerene), respectively. The electrical and optical properties of these materials are grouped in Table 1 [11,15,30,37,38,39,40].

In the aim of validating the actual simulation, a preliminary comparison of the J-V characteristics was carried out between results obtained using the SCAPS simulation and those obtained experimentally [30].

**Figure 2:**
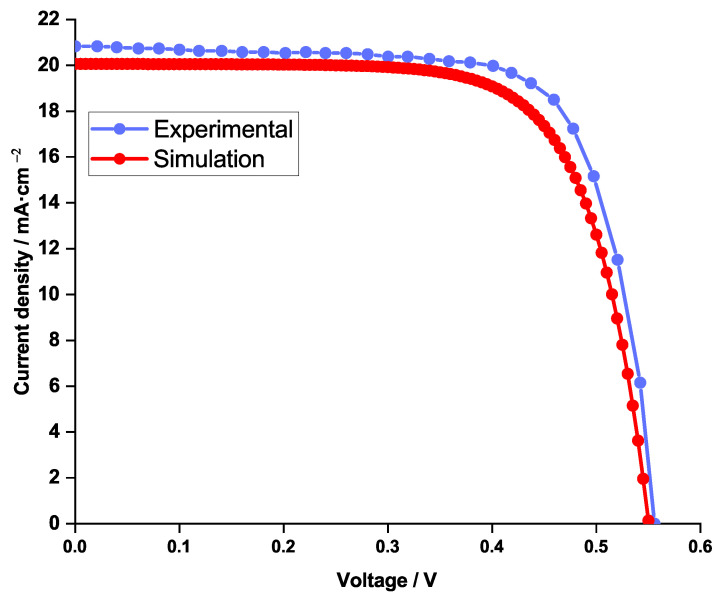
Current density (J-V curve) comparison between simulation (in red) and experimental (in blue) work.

The two curves plotted in Figure 2 are almost superimposed, which proved the effectiveness of the SCAPS simulation. The latter study gave the following cell performance parameters:

- Short-circuit current density $J_{sc}=20.06\;$mA/cm${}^{2}$;
- Open-circuit voltage $V_{oc}=0.55\;$V;
- Fill factor FF=71.64%;
- Power conversion efficiency PCE=7.99%.

To enhance the performance of the PSC, two studies were conducted. The first study consists of testing three other HTL materials: Cu${}_{2}$O (copper oxide), CuSCN (copper thiocyanate) and CuI (copper iodide); and comparing them to the HTL of the initial reference structure (PEDOT:PSS). The parameters of these three HTL materials are classified in Table 2.

The second stage of the study consists of testing four ETL materials: TiO${}_{2}$ (titanium dioxide), ZnOS (zinc oxysulphide), WS${}_{2}$ (tungsten disulfide) and SnO${}_{2}$ (tin oxide); and comparing them to the ETL of the initial reference structure (C60). Table 3 lists the parameters of these four ETL materials.

Table 4 presents the defect densities of the HTL, absorber, ETL, in addition to the interfaces between them (HTL/absorber and absorber/ETL), values commonly used in the literature [43,46,47,48,49,50,51,52,53,54,55].

In addition to the previously mentioned studies on varying the HTL and ETL materials, different parameters such as thickness and the doping concentration of all the PSC layers (HTL, ETL, and absorber) have been modified to study their impact on the device performance in order to ultimately obtain the optimal cell structure.

## 3. Results and Discussion

In this part, the results are presented. First, a study on the HTL material and its effect on the performance of the PSC was performed. As a conclusion of this study, the optimal HTL material was chosen and further investigations were based on it. Second, the effect of ETL material on the solar cell performance was explored. Ultimately, after finding the optimal ETL and HTL material, an investigation on the effect of the absorber material on the performance of the device was conducted.

### 3.1. Effect of HTL Material on the Solar Cell Performance

In solar cell applications, the HTL is found to have a significant influence on its stability and cost. In addition, HTL directly affects light harnessing, carrier extraction and transportation, and perovskite crystallization [56]. Despite their weak stability, organic materials are in general used as HTLs. This instability originates from the changing of organic materials morphology under thermal conditions leading to a change in their properties. An efficient way to overcome the HTL instability is the replacement of organic materials by p-type inorganic materials. In addition, when the thickness of organic HTL layer increases, the resistance increases which further lowers the performance of the cell [57].

Additionally, organic HTL suffer from low hole mobility and low conductivity. To overcome this issue and for an optimal device performance, additives and dopants must be incorporated in the HTL. However, these additives increase the manufacturing cost and can accelerate the degradation of perovskite films [58].

**Figure 3:**
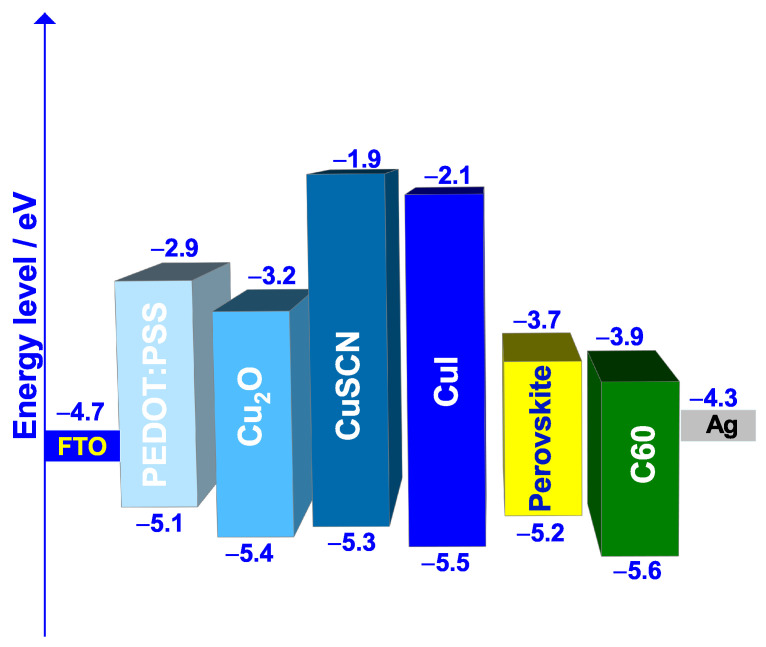
Band alignment between HTL materials and E1G20 perovskite.

Moreover, it has been found from previous works that HTL/absorber and absorber/ETL interfaces play a crucial role in determining the PSC overall performance. In fact, one of the challenges in PSCs is the recombination loss that occurs across the interfaces, especially at the absorber/ETL interface, which can lower the voltage [59]. Therefore, interface engineering and control is highly important in determining the PSC overall performance since the charge injection and the recombination are directly dependent on the properties of both HTL and ETL materials [52,53,60]. To schematize the band alignment between the HTL and the absorber, Figure 3 represents the energy levels of reference and the three tested HTL materials along with the rest of the cell layers.

For a better description of the energy band alignment, the valence band offset (VBO) can be calculated (Equation (4)) using the values of Table 1 and Table 2 or Figure 3.

(4) $$VBO=\chi_{HTL}-\chi_{E1G20}+E_{gHTL}-E_{gE1G20}$$

The values of the VBO of the reference HTL material (PEDOT:PSS) and that of 3 tested HTL materials are grouped in Table 5.

It can be noticed from Table 5 that the HTL used in the reference cell (PEDOT:PSS) has a VBO of −0.1 eV. In fact a negative VBO indicates that a cliff is formed at the HTL/absorber interface, leading to an increasing in the electron-hole recombination rate; therefore causing V${}_{oc}$, J${}_{sc}$, FF and the PCE to monotonically decreases. Conversely, a positive VBO less or equal than 0.3 eV (which is the case with CuI) is found to give excellent J-V characteristics [61,62].

**Figure 4:**
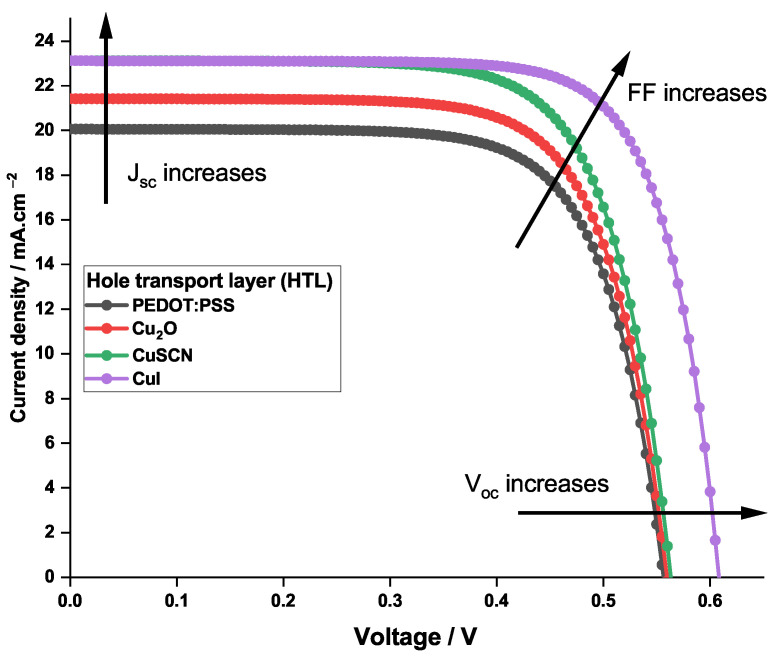
Current density—voltage characteristics for the PSCs with different HTL materials.

To further investigate the effect of the HTL on the performance of the PSC, four different parameters were calculated: the current density, the short-circuit current density, the fill factor, and the voltage open circuit. Figure 4 illustrates the obtained behavior of the above-mentioned parameters for the different HTL materials tested in this work.

The photovoltaic properties obtained using the different HTL materials are listed in Table 6.

**Figure 5:**
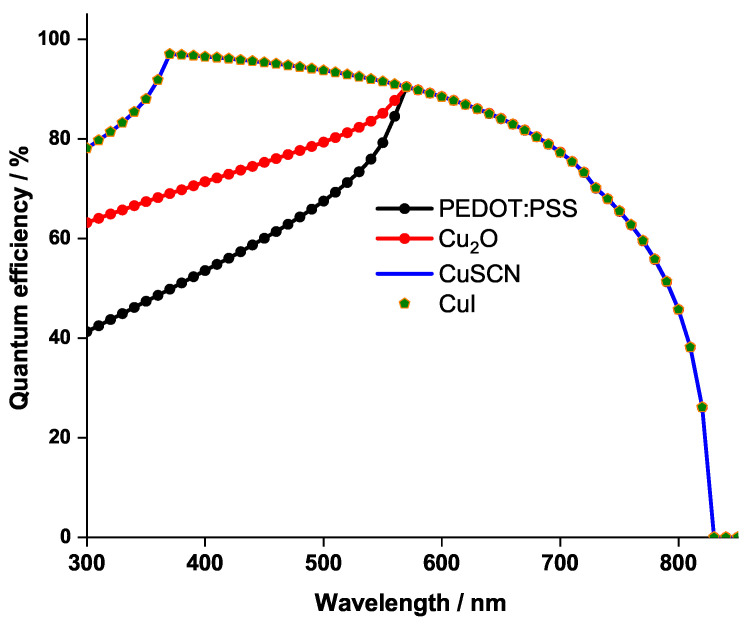
Quantum Efficiency for the PSCs with different HTL materials.

It can be noticed in Table 6 that the fill factor is increasing. In fact, FF is the measure of squareness of the *I*-*V* curve; thus, when the latter squareness increases, as shown in Figure 4, the fill factor increases. Simultaneously, V${}_{oc}$ increases due to the decreasing of charge recombination, leading to the decreasing of the saturation current I${}_{0}$, which in turn increases FF. Moreover, J${}_{sc}$ of CuSCN and CuI is at its highest value, this is due to the large bandgap that these materials have (3.4 eV). In fact, when its bandgap is greater than 2.9 eV, the HTL layer exhibits a better transparency to visible light. Thus, more light will reach the perovskite (E1G20 in this work) layer and will be absorbed, generating more electrons. To confirm this fact, the quantum efficiency for the PSC is represented in Figure 5, for the different HTLs.

The graph shows that only when the light wavelength exceeds 570 nm do the four HTLs have identical quantum efficiency behavior. However, for the lower wavelengths of the visible light spectrum it is clear that only CuI and CuSCN, exhibiting a better transparency, induce high quantum efficiency. The identical behavior of CuI and CuSCN curves is attributed to the fact that the two materials possess the same bandgap energy (Table 2).

The ensemble of the results presented in this section shows that the leading HTL among the tested materials are CuSCN and CuI. Further investigations on the thickness and the doping concentration of the HTL materials are conducted.

#### 3.1.1. Impact of HTL Thickness

The previously presented results on the different HTLs were carried-out for a unified thickness of 100 nm. Previous works [56,63] emphasized the impact of the HTL thickness on the PSC performance. Actually, a thin HTL layer (<50 nm) does not cover completely the absorber layer. Conversely, in the case of a thick HTL layer, chances of recombination are high due to the increasing of the path length of charge carriers and the electric resistance of the device. It is then important to carefully control the thickness of HTL to obtain a full coverage of the rough perovskite layer, without increasing the series resistance of the devices. Hence, a study aiming at finding the best thickness of the HTL was conducted.

**Figure 6:**
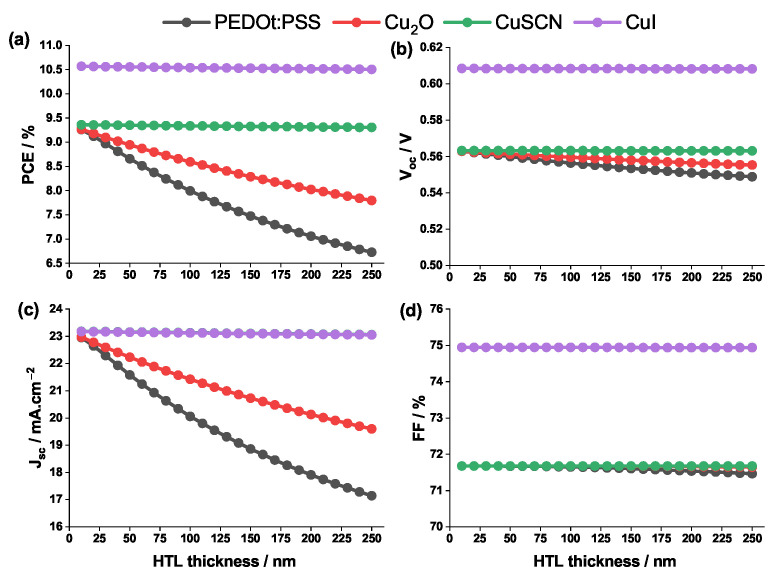
Change in (**a**) PCE, (**b**) V${}_{oc}$, (**c**) J${}_{sc}$, (**d**) and FF against the HTL thickness for different HTL materials.

Figure 6 sketches the change in the photovoltaic properties of PSC as function of the HTL thickness.

As shown in Figure 6, when the thickness is increased from 10 to 250 nm, the four photovoltaic properties remain almost constant for CuSCN and CuI. This is due to the fact that these HTLs have relatively high value of charge mobility (Table 2) and therefore a better conductivity. For the PEDOT:PSS and Cu${}_{2}$O, the larger the value of the thickness the less the value of J${}_{sc}$. In fact, for HTLs with low charge carrier mobility and conductivity (which is the case for PEDOT:PSS and Cu${}_{2}$O), the more the layer thickness increases the more the resistance increases, which further lowers the performance of the cell.

A general analysis of the four graphs in Figure 6 shows that, regardless of its thickness, CuI possesses better properties compared to the other HTL materials. Thus, the optimal HTL material that will be adopted in the remainder of the study, is CuI with a balanced thickness of 100 nm.

#### 3.1.2. Impact of HTL Doping Concentration

In addition to the importance of finding the perfect HTL material and its adequate thickness, it is also crucial to find a balanced value of the acceptor doping concentration (N${}_{A}$). In the previously presented results, the value of N${}_{A}$ was fixed to $1\times 10^{18}$ cm${}^{-3}$ for all the tested HTLs. This part focuses on finding the optimal N${}_{A}$ for CuI HTL material.

**Figure 7:**
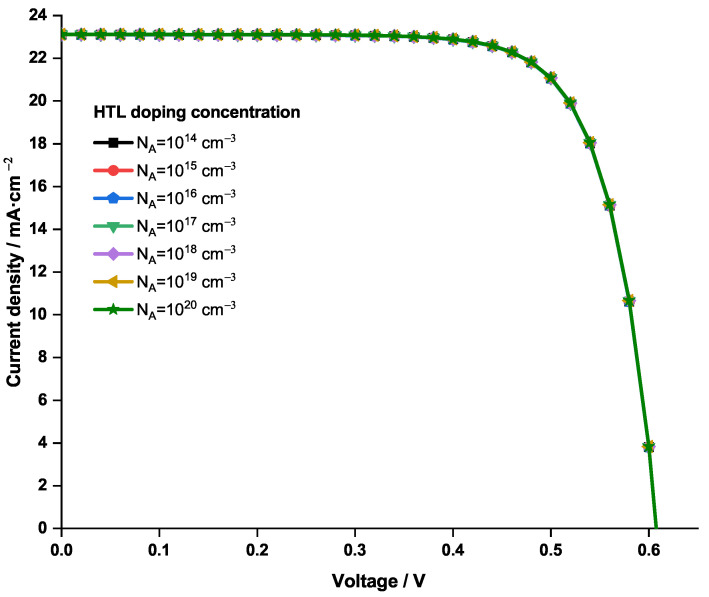
Variation of the current density—voltage characteristics of the PSC with CuI as HTL as function of the acceptor doping concentration.

N${}_{A}$ was varied from $10^{14}$ to $10^{20}$ cm${}^{-3}$; its effect on the current density—voltage curve is plotted in Figure 7.

One can notice from Figure 7 that the obtained current density—voltage curves are overlapping regardless of the value of N${}_{A}$. In fact, previous studies [64,65] suggest that an increasing of the HTL doping concentration causes the fill factor to increase, until a certain upper limit that it cannot exceed. This situation originates from a saturation in the sheet resistance and the conductivity of HTL. In the present case, it is believed that, due to the high conductivity of CuI, this saturation is already attained, even at the lower values of doping concentration. Therefore, no further enhancement on the doping concentration was possible, and the original value of N${}_{A}=1\times 10^{18}$ cm${}^{-3}$ was conserved.

### 3.2. Effect of ETL Material on the Solar Cell Performance

In addition to the importance of adopting a proper HTL material along with its properties, the choice of the electron transport layer (ETL) material is also of high importance. In this work, four different ETL materials were tested and compared to the ETL of the reference structure (C60). The electrical and optical properties of the ETL materials are grouped in Table 3.

Electron transport layer is a major contributor to the interface modification, controlling the charge recombination rates and also aligning the inter-layer energy levels. Moreover, the ETL plays an important role in conducting electrons and blocking holes. Thus, it should have a good conductivity and an excellent band alignment with the perovskite.

**Figure 8:**
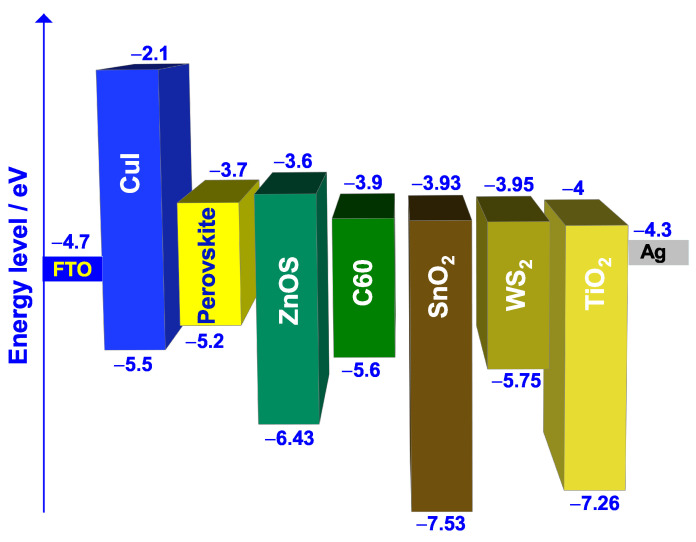
Band alignment between ETL materials and E1G20 perovskite.

The band alignment between the absorber and the ETL is represented in Figure 8.

To better compare the different ETL materials in terms of energy band alignment with the absorber, the conducting band offset (CBO), which is the electron affinity difference between the absorber (E1G20) and the ETL, can be calculated using Equation (5).

(5) $$CBO=\chi_{E1G20}-\chi_{ETL}$$

In general, a positive CBO indicates a spike structure formed at the ETL/absorber interface preventing electrons from reaching the interface. This barrier endowed enhanced photo-generation of free charge carriers, and would suppress the recombination rate at the interface resulting in reducing the V${}_{oc}$. In contrast, a negative CBO indicates that the conducting band level of ETL is lower than that of the absorber, resulting in the formation of an energy cliff at the ETL-perovskite interface.

The values of the CBO of the reference ETL material (C60) and that of the 4 tested ETL materials are grouped in Table 7.

**Figure 9:**
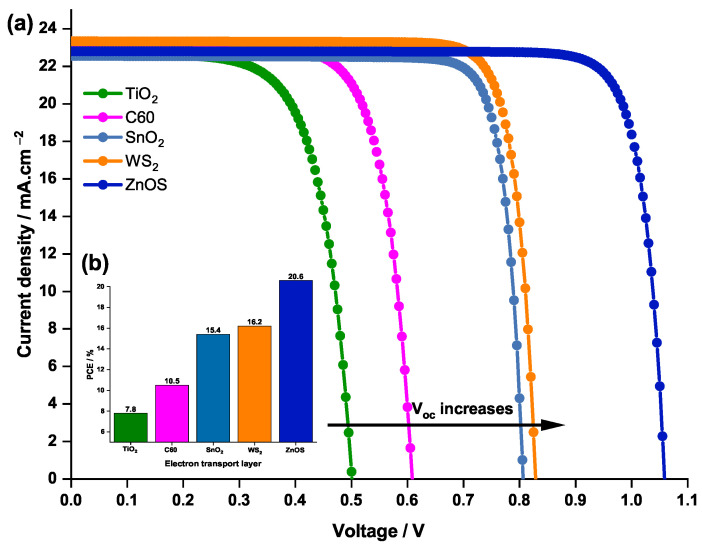
Effect of the ETL material on (**a**) the current density—voltage characteristics of the PSC and (**b**) the PCE.

To further compare the ETLs, the effect of the ETL material on the current density and the voltage characteristics on one hand, and the PCE on the other hand, are sketched in Figure 9.

Figure 9 shows that TiO${}_{2}$, exhibiting the lowest negative CBO, has the lowest V${}_{oc}$, hence its lowest performance with a PCE of only 7.8%.

In parallel with the CBO, another factor that could be the reason behind the difference in behavior between the ETLs is the ETL to absorber dielectric permittivity ratio. This relative permittivity is usually not considered as a design parameter when searching for novel combinations of heterojunction materials for solar cells. However, it was demonstrated by Crovetto et al. [66] that this relative permittivity could have a major influence on the achievable conversion efficiency. In particular, it was found that high-permittivity materials are always the preferred choice as heterojunction partners of the absorber layer when testing new material combinations. This preference is related to the fact that a high ETL to absorber dielectric permittivity ratio makes a solar cell more robust against non-idealities, such as low ETL doping, large required buffer thickness, non-perfect band alignment, interface recombination, or negative interface charge.

In this study, in line with the values of the CBO, the reference ETL (C60) is found to induce a PCE of 10.5% which is a relatively a better performance compared to that with TiO${}_{2}$ (PCE=7.8%). When comparing C60 ETL to SnO${}_{2}$ and WS${}_{2}$ in terms of electron mobility, conductivity, and relative permittivity it is found that these two materials provide better performance than the reference ETL. The latter conclusion is confirmed by the PCE behavior.

The obtained CBO values of all ETL materials are listed in Table 7. It can be noticed that only the ZnOS provide a positive CBO of 0.1 eV (less than 0.2 eV) compared to the other ETL materials where the CBOs are negative. The positive CBO indicates the presence of a spike structure at the interface, leading to a stronger band bending in the conduction band of the ETL, which can prevent the injected electrons from approaching the interface. As a result, this will reduce the charge recombination rate at the interface and increase the V${}_{oc}$ [67], which makes ZnOS the top performing material compared to the other competitors.

The curve plotted in Figure 9 confirms the latter result, where it can be noticed that ZnOS surpasses the other ETL materials, with higher V${}_{oc}$ and PCE (20.6%) values. In conclusion, ZnOS was selected as the optimal ETL and further investigations on this material were conducted.

#### 3.2.1. Impact of ETL Thickness

The previous comparison between the ETL materials was conducted while fixing a layer thickness of 50 nm. In this section, the effect of thickness of the ETL on the performance of the solar cell is investigated.

**Figure 10:**
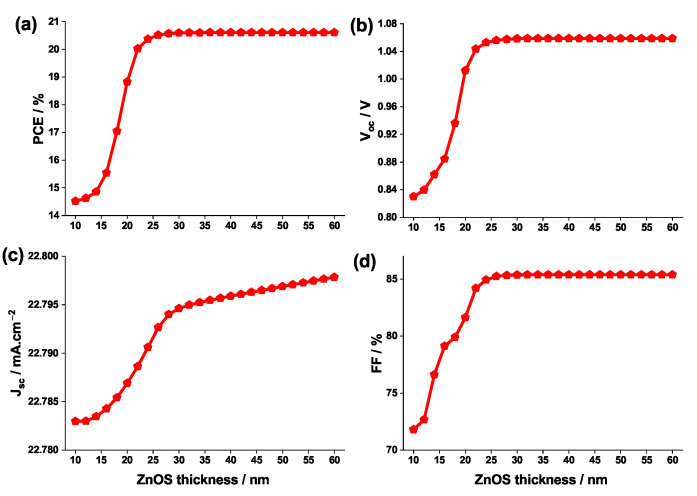
Change in (**a**) PCE, (**b**) V${}_{oc}$, (**c**) J${}_{sc}$, (**d**) and FF as function of the ETL ZnOS thickness.

Figure 10 depicts the evolution of the photovoltaic properties with the ETL ZnOS thickness. According to the latter figure, the PCE, V${}_{oc}$, J${}_{sc}$ and FF all increase rapidly when the thickness varies between 10 and 50 nm, before reaching a maximum of 20.6%, 1.06 V, 22.8 mA.cm${}^{-2}$ and 86%, respectively; afterwards, the PCE, V${}_{oc}$, and FF, exhibit a steady state behavior in the vicinity of the 40–50 nm thickness interval, which can be noticed in Figure 10. Conversely, J${}_{sc}$ increases so negligibly with the thickness, that no plateau is visible. Thus, all the device parameters, except J${}_{sc}$, saturate almost completely for an ETL thickness of roughly 50 nm.

To complement the above analysis, the effect of having an inverted PSC structure should be discussed. This type of structure prevents the ETL material from obstructing the light that is incident from the absorption layer; this allows for the light-induced carriers to be effectively collected and a great number of electron-hole pairs to be excited. At this stage, the thickness has little effect on the device’s J${}_{sc}$ and therefore the ETL absorbs the intensity of the light emanating from the perovskite layer to the greatest extent. Furthermore, increasing the ETL thickness causes the formation of larger pinholes and an uneven surface; this leads to a drastic decrease in the J${}_{sc}$ and V${}_{oc}$, and to a deterioration in the overall efficiency of the PSC [68]. Additionally, a larger thickness, tends to increase the series resistance of the PSC and provokes a drop in J${}_{sc}$ [31,69]. Therefore, the optimal ETL thickness is considered to be 50 nm.

#### 3.2.2. Impact of ETL Doping Concentration

In addition to finding the optimal ETL material (ZnOS) and its adequate thickness (50 nm), the doping concentration (N${}_{D}$) of the ETL material could also have an impact on the J${}_{sc}$ of the solar cell and thus on its PCE. In the previous section the ETL doping concentration was fixed to $N_{D}=1\times 10^{17}$ cm${}^{-3}$ for all the tested materials. In this part, the study of the impact of the ZnOS doping concentration on the PCE and the J${}_{sc}$ is presented.

**Figure 11:**
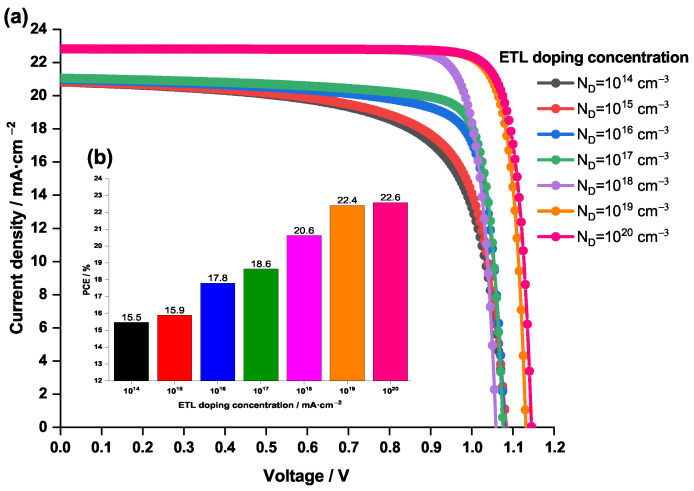
Effect of the doping concentration of the ETL ZnOS on (**a**) the current density—voltage characteristics of the PSC and (**b**) the PCE.

Figure 11 represents the effect of the doping concentration of the ETL ZnOS on the current density—voltage characteristics and the PCE of the PSC; where N${}_{D}$ is varied from $1\times 10^{14}$ to $1\times 10^{20}$ cm${}^{-3}$.

The latter figure shows clearly that the increasing of N${}_{D}$ leads to a rise in both PCE and the J-V characteristic and therefore improving the efficiency of the PSC. In fact, a high doping concentration of the ETL increases the electron conductivity and lower the resistance to the flow of electrons incident from the absorber layer. In addition, previous work done by Xu et al. [70] showed that a high doping concentration of ETL produces deep energy levels at the heterojunction interfaces reducing the non-radiative recombination at the interface and enhancing the cell performance. Moreover, at high doping concentrations, a considerable electric field is produced, which effectively collects the electrons and repels the minority carriers away from the ETL/perovskite interface, thus diminishing interface recombination rates [70]. However, a very high doping concentration of the ETL, of $1\times 10^{20}$ cm${}^{-3}$ and beyond, is generally avoided due to its complex manufacturing process [40].

From Figure 11, it can be noticed that the highest value of $N_{D}=1\times 10^{20}$ cm${}^{-3}$, generates the top performance (in terms of PCE and J${}_{sc}$). In addition, for $N_{D}=1\times 10^{19}$ cm${}^{-3}$, the figure shows that the J-V curve is almost superimposed with that of the $N_{D}=1\times 10^{20}$ cm${}^{-3}$, while the PCE only differs by 0.2%. Therefore, taking into account the above-mentioned disadvantage of raising the N${}_{D}$ rate, the optimum value of ETL doping concentration is suggested to be $1\times 10^{19}$ cm${}^{-3}$.

### 3.3. Effect of the Absorber on the Solar Cell Performance

In addition to the importance that the HTL and the ETL materials add to the PSC performance, the absorber layer makes a major contribution to the solar cell performance. This section is dedicated to the study of the absorber material and its effect on the solar cell performance. In particular the the absorber thickness and its doping concentration are considered.

#### 3.3.1. Impact of the Absorber Thickness

The thickness of the light-absorbing layer was found to be of high importance to the solar cell performance [54,55]. A large value of the thickness increases the current density, but lowers the reverse saturation current, all while increasing the fabrication cost. Moreover, a study done by Bag et al. highlighted that the relation between photon absorption and carrier collection is reciprocal. This study suggests that, when a thin absorber layer (of 200 nm) is used, the obtained photogenerated current will be low, but the charge extraction will be high indicating a decreasing in the recombination rate [64].

In this part, the study of the impact of absorber layer thickness on device photovoltaic outputs is performed. The thickness of the studied absorber material, GAFASnI${}_{3}$, is varied from 100 to 1000 nm while maintaining constant all the other parameters given in Table 2 and Table 4.

**Figure 12:**
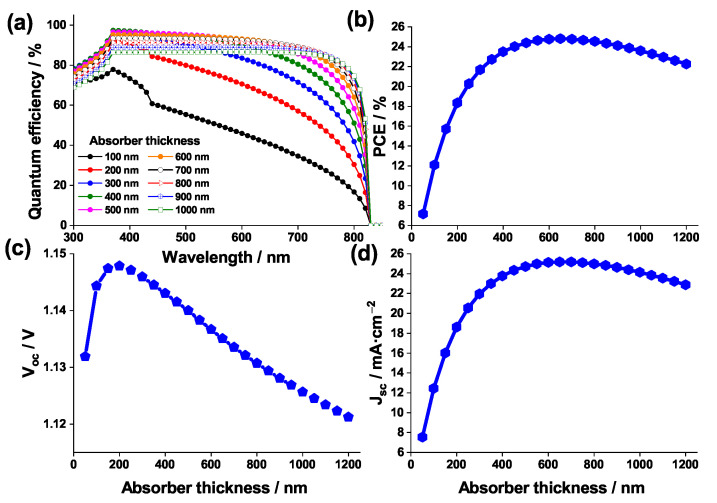
Effect of the absorber thickness on (**a**) the quantum efficiency of the PSC, (**b**) PCE, (**c**) V${}_{oc}$, and (**d**) J${}_{sc}$.

Figure 12 schematizes the effect of the absorber thickness on: quantum efficiency (with respect to the wavelengths of the light), PCE, V${}_{oc}$, and J${}_{sc}$.

The quantum efficiency behavior (Figure 12a) suggests that, in accordance with [71], when the absorber thickness is low, absorption of long wavelength photons is weak. Reversely, when the absorber thickness is high, long wavelength photons are absorbed due to a decrease in the effective band gap. This leads to an improved matching with the solar spectrum [72].

The variation of V${}_{oc}$ is negligible and almost independent of the thickness. However, J${}_{sc}$ increases rapidly from 8 to 25 mA·cm${}^{-2}$ when the thickness is raised from 50 to 600 nm, leading to a similar behavior for the PCE (from 7% to 25%). Above a 600 nm thickness, a drop in J${}_{sc}$ and PCE is visible. This result is due to an enhancement in light absorption, in consistence with the quantum efficiency behavior which reaches its maximum when the thickness is 600 nm; then it starts decreasing beyond this thickness mainly due to the layer resistance increasing when the thickness is high.

As a conclusion, for the remainder of the study the 600 nm thickness is adopted.

#### 3.3.2. Impact of the Absorber Doping Concentration

An additional essential step to choosing the best adsorption thickness layer, is also crucial to determine the adequate acceptor doping concentration of the absorber, aiming to maximize the efficiency of the PSC.

**Figure 13:**
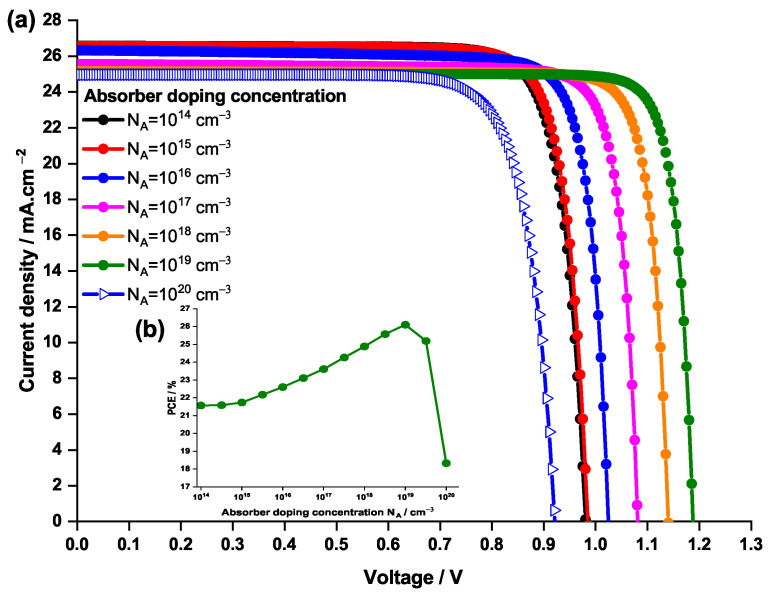
Effect of the doping concentration of the absorber on (**a**) the current density—voltage characteristics of the PSC and (**b**) the PCE.

In this section, the acceptor doping concentration of the absorber has been varied from $10^{14}$ to $10^{20}$ cm${}^{-3}$ and its effect on the PSC properties have been assessed, in particular on the J-V curve and the PCE. The results of this study are schematized in Figure 13. It is to be noted that the initial value of the absorber doping concentration was $10^{17}$ cm${}^{-3}$.

From Figure 13, it can be noticed that the PCE and the J-V curves follow the same trend when the N${}_{A}$ is varied.

One can notice that the PCE significantly increases with the N${}_{A}$, reaching 26% at $N_{A}=10^{19}$ cm${}^{-3}$, showing an improvement compared to the initial case generating a PCE of 23.5%. In fact, when the acceptor doping concentration increases, the Fermi energy level of the hole decreases and hence the V${}_{oc}$ increases. In addition, as shown by Wei Liu et al. [73,74], doping the absorber layer can reduce the trap-state density and increase the carrier lifetime in PSC, which in turn increases the V${}_{o}c$. Furthermore, increasing the doping concentration, leads to increasing the built-in electric field, which results in separation of charge carriers and hence reducing the charge recombination. As a result, this will improve the V${}_{oc}$ and the solar cell efficiency.

However, for values of N${}_{A}$ higher than $10^{19}$ cm${}^{-3}$ it can be noticed that the PCE abruptly decreases to 18.3%; this occurs when the N${}_{A}$ is raised to $10^{20}$ cm${}^{-3}$. This drop is caused by an increasing in the scattering creating a deficiency in carriers collection followed by a significant raise in recombination rates, resulting a drop in the solar cell performance.

Therefore, an $N_{A}=10^{19}$ cm${}^{-3}$ is found to be the optimal value.

## 4. Conclusions

Although an experimental study was completed on a GA${}_{0.2}$FA${}_{0.78}$SnI${}_{3}$-1% EDAI${}_{2}$-based solar cell, the maximum PCE reached remained below 9%. In this paper, an inverted *p*-*i*-*n* structure of HTL/absorber/ETL was investigated using SCAPS-1D simulation software. Four HTL and five ETL materials were comparatively studied, while keeping the remaining layers fixed. The simulation results revealed that an appropriate choice of the ETL and the HTL materials could increase the PCE of the cell to 26%. The solar cell with CuI as HTL surpasses the other HTL materials, owing this to the excellent band alignment with the absorber, its larger band gap, hence its improved transparency to visible light and its high conductivity. Benefiting from the reduced interfacial charge recombination due to the excellent band alignment with the absorber, and the high permittivity, the device with ZnOS as ETL scores a PCE as high as 20.6%, while the reference device with C60 as ETL (used in the experimental work) shows a lower PCE of only 10.5%. The thickness and the doping concentration of all layers have been varied to optimize the photovoltaic performance of CuI/absorber/ZnOS solar cell. The thickness and the doping concentration of the CuI as HTL material have no effect due to their high conductivity. On the other hand, increasing the absorber thickness to 600 nm, and increasing the doping concentration of the ETL and the absorber to $10^{19}$ cm${}^{-3}$ greatly improves the efficiency of the solar cell to reach a record of PCE=26%. The findings of the simulation clearly show the possibility of manufacturing a stable and highly effective hybrid tin-based perovskite solar cell.

## Figures and Tables

**Table 1:** Electrical and optical properties used in simulation of GAFASnI${}_{3}$-based perovskite solar cell.

Parameters	E1G20 [15,30,38,39,40]	PEDOT:PSS (HTL) [11,30,37]	C60 (ETL) [11,30,37]
Thickness/μm	0.35	0.1	0.05
Bandgap E${}_{g}$/eV	1.5	2.2	1.7
Electron Affinity χ/eV	3.7	2.9	3.9
Dielectric permittivity	8.2	2.3	4.2
CB effective density of states/cm${}^{-3}$	$1\times 10^{18}$	$2.2\times 10^{18}$	$8\times 10^{19}$
VB effective density of states/cm${}^{-3}$	$1\times 10^{18}$	$2.8\times 10^{18}$	$1\times 10^{20}$
Electron mobility/cm${}^{2}$/V·s	22	$2\times 10^{-4}$	0.08
Hole mobility/cm${}^{2}$/V·s	22	0.02	$3.5\times 10^{-3}$
Donor Concentration N${}_{D}$/cm${}^{-3}$	0	0	$1\times 10^{17}$
Acceptor concentration N${}_{A}$/cm${}^{-3}$	$1\times 10^{17}$	$1\times 10^{18}$	0

**Table 2:** Electrical and optical properties of different HTL materials.

Parameters	Cu${}_{2}$O [41,42]	CuSCN [43,44]	CuI [43,45]
Thickness/μm	0.1	0.1	0.1
Bandgap E${}_{g}$/eV	2.170	3.4	3.4
Electron Affinity χ/eV	3.2	1.9	2.1
Dielectric permittivity	7.11	10	10
CB effective density of states/cm${}^{-3}$	$2.02\times 10^{17}$	$2.2\times 10^{18}$	$1\times 10^{18}$
VB effective density of states/cm${}^{-3}$	$1.1\times 10^{19}$	$1.8\times 10^{19}$	$1.8\times 10^{19}$
Electron mobility/cm${}^{2}$/V·s	200	100	2$\times 10^{4}$
Hole mobility/cm${}^{2}$/V·s	80	25	2$\times 10^{4}$
Donor Concentration N${}_{D}$/cm${}^{-3}$	0	0	0
Acceptor concentration N${}_{A}$/cm${}^{-3}$	$1\times 10^{18}$	$1\times 10^{18}$	$1\times 10^{18}$

**Table 3:** Electrical and optical properties of different ETL materials.

Parameters	TiO${}_{2}$ [46,47]	ZnOS [43]	WS${}_{2}$ [48,49]	SnO${}_{2}$ [50,51]
Thickness/μm	0.05	0.05	0.05	0.05
Bandgap E${}_{g}$/eV	3.26	2.83	1.80	3.60
Electron Affinity χ/eV	4	3.60	3.95	3.93
Dielectric permittivity	32	9	13.6	8
CB effective density of states/cm${}^{-3}$	$1\times 10^{19}$	$2.2\times 10^{18}$	$2.2\times 10^{18}$	$3.1\times 10^{18}$
VB effective density of states/cm${}^{-3}$	$1\times 10^{19}$	$1.8\times 10^{19}$	$1.8\times 10^{18}$	$2.5\times 10^{19}$
Electron mobility/cm${}^{2}$/V·s	20	100	100	15
Hole mobility/cm${}^{2}$/V·s	10	25	100	0.1
Donor Concentration N${}_{D}$/cm${}^{-3}$	$1\times 10^{17}$	$1\times 10^{17}$	$1\times 10^{17}$	$1\times 10^{17}$
Acceptor concentration N${}_{A}$/cm${}^{-3}$	0	0	0	0

**Table 4:** Defect density values inside the layers and at interfaces of the device.

Parameters	HTL	ETL	E1G20	HTL/E1G20	E1G20/ETL
Defect Type	Neutral	Neutral	Neutral	Neutral	Neutral
Capture cross section for electrons $\sigma_{n}$/cm${}^{-2}$	$1\times 10^{-15}$	$1\times 10^{-15}$	$1\times 10^{-15}$	$1\times 10^{-18}$	$1\times 10^{-15}$
Capture cross section for holes $\sigma_{p}$/cm${}^{-2}$	$1\times 10^{-15}$	$1\times 10^{-15}$	$1\times 10^{-15}$	$1\times 10^{-16}$	$1\times 10^{-15}$
Energetic distribution	Single	Single	Gaussian	Single	Single
Energy level with respect to E${}_{v}$ (above E${}_{v}$)/eV	0.650	0.65	0.6	0.6	0.6
Characteristic energy/eV	0.1	0.1	0.1	0.1	0.1
Total density N${}_{t}$/cm${}^{-3}$	$1\times 10^{15}$	$1\times 10^{15}$	$1\times 10^{15}$	$1\times 10^{12}$	$1\times 10^{12}$

**Table 5:** Valence band offset for the HTL materials.

HTL	VBO/eV
PEDOT:PSS (Reference cell)	−0.1
Cu${}_{2}$O	0.2
CuSCN	0.1
CuI	0.3

**Table 6:** Photovoltaic properties obtained using the different HTL materials.

HTL	J${}_{\mathrm{sc}}$/mA/cm${}^{2}$	FF/%	V${}_{\mathrm{oc}}V$	PCE/%
PEDOT:PSS (Reference cell)	20.60	71.64	0.556	7.9973
Cu${}_{2}$O	21.42	71.72	0.559	8.5800
CuSCN	23.13	71.78	0.560	9.3525
CuI	23.12	74.94	0.600	10.5400

**Table 7:** Conducting band offset and ETL/absorber permittivity ratio for the ETL materials.

ETL	CBO/eV	ETL/Absorber Permittivity Ratio
C60 (Reference cell)	−0.2	0.512
SnO${}_{2}$	−0.23	0.975
TiO${}_{2}$	−0.3	3.9
ZnOS	0.1	1.09
WS${}_{2}$	−0.25	1.67

## Data Availability

Not applicable.

## Abbreviations

The following abbreviations are used in this manuscript:

PSC	perovskite solar cell
PCE	power conversion efficiency
V${}_{Sn}$	reduced formation energy of Sn vacancies
E1G20	GA${}_{0.2}$FA${}_{0.78}$SnI${}_{3}$-1% EDAI${}_{2}$
HTL	hole transport layer
ETL	electron transport layer
V${}_{oc}$	open circuit voltage
J${}_{sc}$	short circuit current density
QE	quantum efficiency
FF	fill factor
FTO	Fluorine-doped tin oxide
VBO	valence band offset
N${}_{A}$	acceptor doping concentration
CBO	conducting band offset
N${}_{D}$	donor doping concentration
